# Latent Autonomic Dysfunction in a Chronic Cervical Spinal Injury

**DOI:** 10.7759/cureus.36785

**Published:** 2023-03-28

**Authors:** Hiroshi Fujioka

**Affiliations:** 1 Department of Neurosurgery, Ohshima Hospital, Saga, JPN; 2 Department of Neurosurgery, Cognitive and Molecular Research Institute of Brain Diseases, Kurume University, Fukuoka, JPN

**Keywords:** chronic disorders, syncope, alcohol, autonomic dysfunction, cervical spinal injury

## Abstract

Latent autonomic dysfunction has been identified in recent years among patients with chronic cervical lesions. This paper further illustrates a precautionary case of symptomatic manifestation with an elusive trigger. A 64-year-old male, who had shown excellent neurological recovery after decompression surgery for a cervical spinal injury (modified Frankel classification from C1 to D3), complained of recurrent syncope in the chronic phase. The cause remained unidentified for two years, but it was finally discovered that the syncope was induced by a transient sympathetic overactivation that was concurrent with mental strain and alcohol intake. Abstinence completely suppressed the episodes thereafter. The case suggests the possibility that patients with a history of cervical spinal injury, no matter how normal they appear, may have asymptomatic autonomic dysfunction. Additionally, identification of the trigger can be challenging due to its dynamic and protean nature. More emphasis should be paid to autonomic evaluation for chronic cervical spinal injuries.

## Introduction

Latent or subclinical autonomic dysfunction has been identified in recent years among patients with chronic cervical lesions [1-3]. Such dysfunction was recognized in cardiovascular systems, including heart rate variability (HRV) changes, decreased blood pressure, and orthostatic hypotension [1-3]. Whereas these cases remained asymptomatic throughout the investigation, the author encountered a precautionary case of symptomatic manifestation due to an elusive trigger, indicating the importance of chronic autonomic evaluation for cervical spinal injuries.

## Case presentation

A 64-year-old male was transferred to the emergency department due to quadriplegia after an accidental fall while walking. His past clinical records indicated hypertension and supraventricular arrhythmia, with no episodes of syncope. Medications included a calcium blocker and an angiotensin receptor blocker. He had a surgical history of cervical herniation at the C6/7 vertebral level a few decades ago, without any resulting neurological or cardiovascular deficits. He was a social drinker.

**Figure 1:**
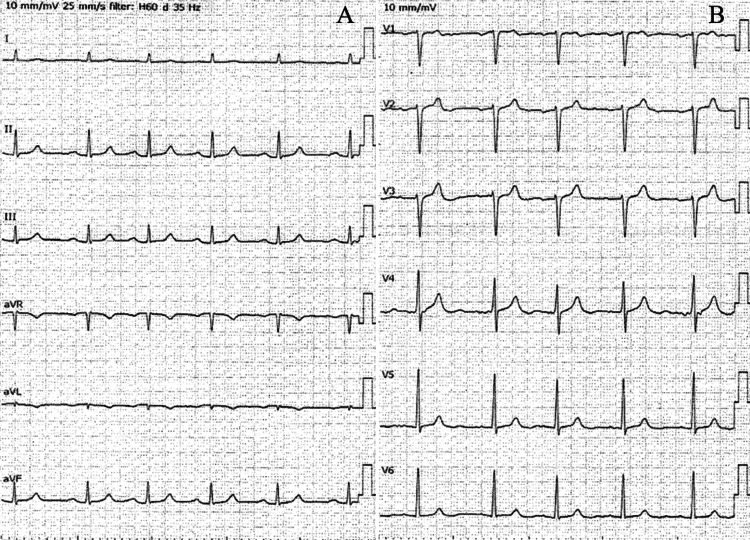
The 12-lead ECG on admission The limb (A) and chest (B) leads indicated a first-degree atrioventricular conduction block.

On admission, blood pressure indicated hypotension (60/45 mmHg) with brady- to normocardia (50-60 bpm). Electrocardiogram (ECG) revealed a first-degree atrioventricular conduction block (Figure 1). Laboratory data were unremarkable. A neurological examination indicated incomplete quadriplegia and sensory deficits below the C5 spinal level without bladder or rectal disturbance (modified Frankel classification C1). Horner’s syndrome was not noted.

**Figure 2:**
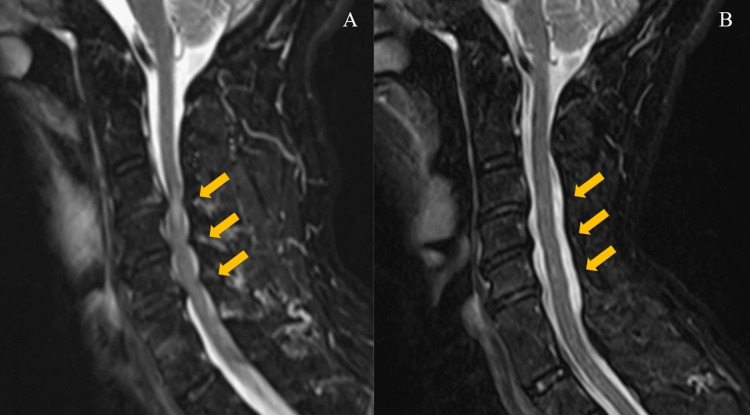
Cervical MRI with short T1 inversion recovery (STIR) sequence Cervical MRI before (A) and after (B) surgery. Decompression surgery (C3-6 laminoplasty (orange arrows) with C2 and C7 dome laminectomy) was performed about one month after admission.

Cervical CT was unremarkable except for a slight hematoma in front of the cervical vertebrae. Instability was not noted. A subsequent cervical MRI indicated signal changes at the C3-7 vertebral levels and canal stenosis at the C3/4, C4/5, and C5/6 vertebral levels (Figure 2A, orange arrows).

Since neurological deficits ameliorated early during hospitalization, conservative therapy with physical rehabilitation was initially chosen. Neurological recovery began to reach a plateau, and cervical laminoplasty (C3-6) was conducted one month after the injury (Figure 2B, C3-6 decompression was indicated by orange arrows). Preoperative cardiovascular investigations were unremarkable, with a blood pressure of 115/65 mmHg, normal ECG, and a left ventricular ejection fraction of 63% based on an echocardiogram.

His postoperative clinical course was excellent, and he was discharged home two months after admission. Neurological deficits at the time of discharge were limited to left shoulder pain and sensory disturbance involving the right leg (modified Frankel classification: D3). Cardiovascular symptoms were stable throughout hospitalization without any changes in medications.

While daily activities increased enough to resume full-time work, he experienced asymptomatic fluctuations of the systolic blood pressure (80-150 mmHg) and heart rate (40-90 bpm). He subsequently experienced two episodes of syncope at business dinners, where he consumed a small volume of alcohol. Each syncope was associated with a prodrome of dizziness and loss of vision, and a marked decrease in the systolic pressure (50-60 mmHg). Differential diagnoses were neurally mediated syncope (orthostatic or vasovagal syncope), cardiac syncope, and complex partial seizures.

Neurological investigations were unremarkable based on head MRI and electroencephalography. An echocardiogram indicated a decrease in the left ventricular ejection fraction of 51%. Holter ECG was unremarkable, with a sporadic premature ventricular complex and supraventricular arrhythmia. HRV indicated a coefficient of variation of the R-R interval of 1.92; < 2.0% is generally used as a threshold for autonomic disturbance [4].

Orthostatic syncope was initially suspected, but orthostatic hypotension (i.e., a decrease in blood pressure of >20 mmHg systolic and/or >10 mmHg diastolic 3 minutes after standing [5]) was not recognized. In addition, syncope was always observed on adopting a sitting position without orthostatic stress. Further autonomic investigation using the head-up tilt test was refused by the patient for fear of iatrogenic syncope. Another strong candidate was alcohol intake [6]; however, a few opportunities to drink alcohol at home did not induce syncope. A small amount of beta-blocker (carvedilol, 2.5 mg/day) was subsequently administered by a cardiologist.

The above cardiovascular disorders gradually stabilized over months, but he still had two additional syncopal episodes two years after the initial onset. The next episode of syncope, which was observed about a half hour after drinking at home, provided an important clue: according to his family, he had felt strong mental stress and fatigue at the time of drinking. Further history-taking revealed that past syncopal events were all associated with the combination of alcohol intake and mental stress, whereby the potential involvement of transient sympathetic overactivation was strongly suspected. Following complete abstinence, syncopal events disappeared, with no event over the last two-year follow-up period.

## Discussion

The heart receives sympathetic innervation from the supraspinal centers via the cervical spinal cord [7]. Based on the anatomy, cervical spinal injuries in the acute phase are often associated with cardiovascular problems [8,9]. Typical observations are hypotension and bradycardia, and, in severe cases, autonomic dysreflexia and cardiac arrest can occur [10]. These autonomic symptoms, if not severe, will recover spontaneously within several weeks [8], and less attention has been given to those in the chronic phase.

Recently, however, latent autonomic dysfunction was identified among patients with chronic cervical lesions [1-3]. Patients with compressive cervical myelopathy, for example, had subclinical autonomic dysfunction based on conventional autonomic function tests and HRV [3]. Supporting the previous findings, the present case further demonstrated a symptomatic manifestation as recurrent syncope with an elusive trigger.

Cervical spinal injuries are sometimes associated with orthostatic syncope or hypotension [9]. Given the transient sympathetic overactivation and vagal overcoming without orthostatic stress, the present case could be regarded as showing vasovagal syncope. Although its pathophysiology is still incompletely understood, vasovagal syncope is considered to be caused by an exaggerated sympathetic response that is followed by vagal overcoming [11].

While mental strain was the primary driving force in the present case, concurrent alcohol intake was necessary for manifestation. It is known that alcohol intake per se can be associated with syncope in the acute phase either through vasovagal [6] or orthostatic [12] syncope. The former may be induced by stimulation of sympathetic activities and inhibition of parasympathetic activities, possibly via the Bezold-Jarisch reflex [6], with the latter caused by orthostatic stress due to impairment of vasoconstriction [12]. Considering that alcohol-induced syncope is enhanced by sympathetic activation factors, such as overwork, dehydration, overheating, and mental stress [6], it could be considered that, under the chronic condition of latent autonomic dysfunction via cervical spinal injury, sympathetic activation was transiently enhanced by mental strain and alcohol intake, which resulted in vasovagal syncope.

Whereas syncope is generally considered benign, it could be associated with the risk of traffic accidents or drowning during bathing. Such a risk might incur more serious consequences in patients with a history of cervical spinal injuries. Identification of the cause is difficult, and so as many as 50-75% of syncopal events remain etiologically unidentified [13]. Vasovagal syncope is protean in nature, and due to the heterogeneity of chronic cervical injuries, the “trigger” that induces syncope may vary in each patient. Furthermore, such a trigger may not be a single factor but a combination of multiple factors. In clinical practice, we must be aware that the reliability of autonomic testing (HRV, Valsalva maneuver, head-up tilt test, etc.) is limited, and that history-taking with the patient is critically important [13].

The therapeutic effects of surgical interventions on autonomic disturbance are currently unclear and even conflicting. The potential effectiveness of decompression surgery was illustrated in one case with autonomic dysreflexia caused by cervical stenosis [14]. The benefits of decompression surgery were further confirmed by one prospective study in patients with latent autonomic dysfunction as well as neurological dysfunction [1]. However, one retrospective study reported that de novo orthostatic hypotension was postoperatively recognized in 11.6% of patients with cervical spine surgery [2]. Considering that no definite therapies for vasovagal syncope are currently available [11], the therapeutic potential of decompression surgery for cervical autonomic dysfunction will be an important issue to be investigated.

## Conclusions

To conclude, our case demonstrated subclinical autonomic dysfunction in a normal-appearing patient with a history of cervical spinal injury, and such dysfunction was symptomatic in the presence of complex factors that may involve transient sympathetic overactivation. Despite the recent attention to autonomic dysfunction after spinal cord injury, knowledge of the mechanisms is still limited; hence, further research is warranted.
